# Contemporary human rights violations in female sterilization care: legal and ethical considerations when coerced patients *do* consent

**DOI:** 10.1007/s40592-025-00240-7

**Published:** 2025-05-13

**Authors:** Liana Woskie, Mindy Jane Roseman

**Affiliations:** 1 Department of Community Health, Tufts University, 547 Boston Ave, Medford, MA 02115 USA; 2 Department of Global Health and Population Health, Harvard T. H. Chan School of Public Health, 677 Huntington Ave, Boston, MA 02155 USA; 3 Gruber Program for Global Justice and Women’s Rights, Yale University School of Law, New Haven, CT USA

**Keywords:** Coercion, Sterilization, Informed consent, Human rights

## Abstract

In this piece we examine three forms of coercive or otherwise involuntary care that can occur *with* patient consent. To do so, we examine: (1) uninformed consent, (2) contingency-based consent and (3) constrained-market consent, amongst female sterilization patients. While there is broad recognition that “coercion” in sterilization care can manifest beyond instances of overt force and clarity on what constitutes coercion within clinical care, this has not translated to accountability. The current practice of identifying coercion through discrete civil cases may facilitate a narrow understanding of its contemporary prevalence; one that does not align with definitions of coercion supported by international human rights entities. We use three acute, and widely recognized, examples—hysterectomies in ICE detention facilities, India’s sterilization camp deaths and birth control quotas for Uyghur women—as an entry point to highlight less overt contemporary forms of coercive sterilization care, pairing each example with data that explores prevalence at a broader population level. These data suggest less visible forms of coercion may persist relatively unchallenged—raising the ethical case for a functional approach to the measurement of coercion. In turn, we argue the relevant question may not be “when is coercion ethically justified in public health,” but rather, why is coercion already the status quo?

## Introduction

Every several years there is a widely publicized instance of egregious (Jha et al. 2015) and rights-violating female sterilization care—in 2014, the death of over a dozen women in India’s sterilization camps, in 2020 unnecessary hysterectomies by the “Uterus Collector” in a rural Georgia ICE detention center (Jennings 2021), more recent reports of Uyghur women undergoing sterilization in China en masse (Waller and Albornoz 2021). The cases have been covered by major media outlets that outline and denounce the use of coercive tactics (Gowen 2014). Public sentiment regarding these cases has been relatively clear: they are unacceptable (Forced 2019). However, they are often considered, and legally treated, as isolated instances experienced by structurally marginalized or detained individuals. The public understanding of coercive sterilization aligns with this characterization—forced sterilization is a historical phenomenon, one that rears its head every so often in discrete circumstances, but is ultimately an issue of the past.

Today, female sterilization (i.e. tubal ligation or hysterectomy) is considered an important and viable form of birth control and contributes significantly to both U.S. and international measures of reproductive care coverage (UNDP 2020). Coerced sterilization, in contrast, is widely recognized as a human rights violation and, when concentrated amongst specific populations, can be considered a form of reproductive genocide (Genocide Prevention and the Responsibility to Protect 2022; Carranza Ko 2020). The principle of autonomy, expressed through full, free and informed decision-making, is a central theme in medical ethics, and is embodied in human rights law (Faden 1986; Resolution on Involuntary Sterilisation and the Protection of Human Rights in Access to HIV Services (Res) 2013). People must be able to choose and refuse sterilization (The WHO 2014). Ensuring free choice requires that the health-care interaction is non-directive—but also meets basic pre-conditions required for an individual to make a choice autonomously (Desjardins 2012; Senderowicz 2020). For sterilization care, these pre-conditions for autonomy are relatively well defined and consistently outlined, for example: ensuring a patient knows the procedure is permanent, is free from incentives or inducement and has access to alternate options, among other basic requirements (The WHO 2014). The absence of these pre-conditions, in turn, is recognized by the United Nations (U.N.) Human Rights Office and other U.N. agencies as coercive or otherwise involuntary (The WHO 2014). In this piece we aim to add specificity to the conversation on coercive public health by examining three clearly agreed-upon, but less overt, forms of coercive sterilization care that can occur within the purview of the health system. We focus on patients who may technically provide “consent” but for whom pre-conditions are not met. In line with U.N. outlined pre-conditions, we categorize these instances as uninformed consent, contingency-based consent, and constrained market consent. While there is consistent recognition that coercion can manifest beyond instances of overt force, this discursive recognition has not translated to how violations are assessed (Senderowicz 2020). We argue that despite clarity on what constitutes coercion—including in less overt and, in turn, visible instances—this has not translated to measurement, compromising accountability.

When coercion in sterilization care comes to public attention exclusively through legal cases in which an overtly egregious situation or rights violation has raised concern, this may lend itself to a relatively narrow understanding of the contemporary prevalence of coercive sterilization care. If this is the case, it does not align with broader definitions supported by international human rights entities. By treating pre-conditions for consent as concrete and measurable phenomena within the coercion discourse, we can inform a more nuanced understanding of contemporary prevalence. We recognize this approach is practical and does not provide a complete examination of coercion—it requires a certain “flattening” of the concept, which is complex. However, by focusing on pre-conditions, we can provide a more concrete assessment of cases in which autonomy could not possibly be met. In taking this approach, we argue that less overt forms of coercion (such as uninformed consent) may persist widely and relatively unchallenged within the routine delivery of care.

## Coercion with consent

### Normative rights

A standard typology to understand influence (and in turn coercion) within the field of bioethics was developed by Ruth Faden, Tom Beauchamp, and Nancy King (Faden 1986). They categorize types of influence into the following: rational persuasion, manipulation, and coercion (Faden 1986). While coercion, or influence by irresistible threats or offers, is widely understood as problematic within public health, there is less clarity regarding the other two categories. Rational persuasion, for example, may not only be ethically permissible but advised when in service of a public good – such as decreasing smoking or uptake of vaccines (Blumenthal-Barby 2012). As Blumenthal-Barby raises, for much of public health, the primary points of contention lie within the category “manipulation.” (Blumenthal-Barby 2012) This includes influence by incentivizing, increasing options, decreasing options, tricking, using [resistible] threats of punishment, managing information, presenting information in a way that leads to predictable inferences, deceiving, withholding information, and exaggerating it in a misleading way (Blumenthal-Barby 2012). At present, sterilization is a relatively unique case in that, acts of manipulation, as outlined by Faden, Beauchamp and King, fall clearly within the summative human rights category “coercive or otherwise involuntary care.”

Historically, a lack of consensus on what constituted “coercion” plagued reproductive care programming within the broader field of public health. The 1994 International Conference on Population and Development in Cairo (ICPD) represented a historical turning point, where there was global demand for an end to family planning programs geared toward population control (United Nations 1995,1994). The resulting Programme of Action called for a broader understanding of sexual and reproductive health that emphasized three pillars: rights, access, and quality within family planning programs (Coomaraswamy 2005). UN Political Conferences, including the Platform for Action of the 1995 Fourth World Conference on Women (Beijing), as well as their subsequent reviews set precedence for defining coercion (United Nations 1995). For example ICPD (and amplified in Beijing) recognized that coercing individuals in matters related to reproduction—including sterilization—violated human rights (United Nations 1995),^1^ At these conferences, States agreed to end population policies carried out through coercion. Corresponding agreements outlined several attributes of non-coercive programming, such as: available, accessible, and affordable reproductive care—staffed by trained professionals who would provide individuals with accurate and quality family planning information and services—an admirable commitment, but sufficiently broad to largely avoid critical assessment (Roseman and Reichenbach 2011).

As Senderowicz writes, the commitments emerging from Cairo have, in practice, remained largely discursive. While the language in reproductive health programming shifted, both issues of rights and quality remain largely absent from contemporary measurement strategies:

“In many ways… the post-Cairo shift toward these pillars has been more successful rhetorically than substantively. While the language used to describe family planning has shifted dramatically in the 25 years since ICPD, changes to how we conceptualize, implement, and evaluate family planning programs have been far less complete. (Senderowicz 2020)

In 2014 the United Nations (U.N.) Human Rights Office and six other UN Agencies co-produced a report: “*Eliminating forced, coercive and otherwise involuntary sterilization*” (The WHO 2014). The report did not address other forms of reproductive care, but provided guiding principles for the prevention and elimination of coercive *sterilization*—as well as an actionable definition of what constitutes coercion. Using this framework, coerced sterilization occurs when financial or other incentives, misinformation, or intimidation tactics are used to compel an individual to undergo the procedure (The WHO 2014). The framework also makes clear that coerced sterilization can occur in the absence of informed consent. Unlike overt or irresistible threats, coercion in this case may include behavior that is positive or potentially unknown as a rights-violation to the patient, such as financial incentivization (The WHO 2014; Freedman et al. 2014). Information that is required for informed consent is clearly outlined and includes factors such as: knowledge that a tubal ligation procedure is permanent, knowledge of alternate family planning option, freedom from misinformation, etc (The WHO 2014). Individuals and societies may have different ideas of how to weigh aspects of the framework, but as a rights-based document with broad multi-lateral support the intent was to provide a context agnostic baseline i.e. any woman in any country should be told that a tubal ligation procedure is permanent prior to undergoing the procedure, regardless of what they choose to do with that information (Woskie 2025).

The framework also aligns with work from Radhika Coomaraswamy, Special Rapporteur on Violence Against Women (SRVAW), to outline the attributes and role of state violence, which may be more pervasive and less overt than other forms of violence (Coomaraswamy 2005; Jewell 2012) In identifying actions that should be taken within the health system—the Interagency Framework situates responsibility at a structural level. This aligns with a longer-standing body of scholarship on structural violence (Nandagiri 2020). A term coined by Johan Galtung during the 1960s, structural violence describes the role of social structures that stop individuals, groups, and societies from reaching their full potential (Galtung 1969). In its general usage, the word “violence” often refers to physical acts; however, Galtung’s scholarship argued violence can extend to the “avoidable impairment of fundamental human needs or…the impairment of human life, which lowers the actual degree to which someone is able to meet their needs below that which would otherwise be possible” (Farmer et al. 2006). In turn, structural violence in healthcare is often embedded in longstanding social structures, normalized by institutions and regular experience (Galtung 1969; Farmer et al. 2006). Because violations are ordinary, they may appear almost invisible. Disparate access to family planning options, restrictions on political office, and legal standing are examples within the context of sterilization care.

The international human rights basis, as well as the bioethical foundations for, informed consent more broadly derive from responses to the Nazi medical experimentation during World War II (Constantin A. Human Subject Research 2018). As articulated in the International Covenant on Civil and Political Rights “No one shall be subjected without his free consent to medical or scientific experimentation” (International Covenant on Civil and Political Rights 1966). A subsequent human rights treaty, the Convention on the Elimination of all forms of Discrimination against Women (Women’s Convention), provided more clarity, recognizing that women “have the right to be fully informed, by properly trained personnel, of their options in agreeing to treatment or research, including likely benefits and potential adverse effects of proposed procedures and available alternatives” (Cole 2016).^2^ Coupled with Article 16(1)(e) of the Women’s Convention—the right to decide freely and responsibly on the number and spacing of children, as well as have access to the information, education, and means to do so— the right to make informed decisions about sterilization, to consent or to refuse, is well supported if not incontrovertible.

In this article, we define situations in which there is invalid consent as tantamount to “no consent.” The absence of affirmative consent, or the ability to meet pre-conditions for consent, in turn constitute coercion. This approach aligns with the U.N. Human Rights Office definition of coercion as articulated for sterilization care. It is a broader and more inclusive definition of coercion than often applied across public health practice but is relevant to how we conceive of and assess less overt, and potentially normalized, forms of coercion.

### Three examples in practice

International human rights laws and norms place an obligation on the public health system to ensure that the rights of individuals to make informed decisions and give (or refuse) consent to reproductive health care, treatment, and services—free from coercion, discrimination, and violence are respected, protected, and fulfilled (on the Right to Sexual and Reproductive Health. United Nations 2016).

Examining instances in which coercion can occur where a patient does provide written or verbal “consent” but in which that consent may be otherwise compromised is a useful entry point into how we operationalize the protection of rights and, in turn, assess coercion within public health practice. This exercise forces a de-coupling of basic concepts. The first category, and term “uninformed consent” indicates clearly that a patient *has* expressed consent but has done so without the baseline information required to do so freely (Category I). The second example “incentivized consent” includes situations in which a patient may have expressed consent, and done so with full information, but there were monetary or non-monetary incentives in place without which they could have made a different decision (Category II). Finally, constrained market consent describes a situation in which a patient could express consent, have full information and be free from incentives—but lack alternate options to curtail fertility (Category III). This final category, in its inverse, may also be referred to as “full choice,” as previously described by Senderowicz. While outlined separately and consecutively, these three categories may (and often do) overlap i.e. a patient may lack basic information as well as alternate options.

More broadly, the three categories are not meant to be comprehensive. Coercion can manifest much more overtly i.e. through physical force or verbal threat, or through social norms—such as deference to clinical providers in the context of power differentials. The three examples were selected for several reasons. First, they fall within the purview of the public health system, in contrast to inter-personal familial or community-based coercion (i.e. where pressure may originate from a spouse or community). Second—they are relatively discrete and can be assessed with objective metrics (i.e. if a financial incentive was provided, if alternate options were made available, etc.). While it is useful to understand if someone *felt* pressured or otherwise coerced, how patients report subjective judgments can vary due to factors external to the clinical interaction, making them potentially more subject to dispute (Woskie 2025). By examining relatively objective aspects of care, the three categories align with international guidance that is normative and meant to be consistent across contexts. For example, all individuals have a right to alternate family planning options, but what individuals ultimately choose to use may differ based on personal preference or community norms. And, finally, each category represents a clear situation in which consent (even if provided) would be considered invalid—i.e. a precondition.

Below we examine each of these categories using a specific, acute case as well as population level data. In each case, there are multiple intersecting factors that may invalidate consent. In each case, there is also a more overtly egregious issue that fueled public attention. We use the more overtly egregious case as an entry-point to examine a less overt, and potentially normalized, form of coercion also present in that case. This approach allows us to explore each normalized form of coercion in the context of a more broadly understood and recognized, surfacing how less overt forms of coercion may manifest within the routine delivery of care. This is an attempt to extend beyond theoretical discussion and examine how rights-based definitions of coercion may be experienced at the population level.

## CASE I: 2020 ICE hysterectomies

The first case we examine is that of hysterectomies conducted in 2020 within a U.S. Immigration and Customs Enforcement (“ICE”) Detention Center run by LaSalle LLC, and resulting in the filing of a resultant legal case, Oldaker v. Giles, in U.S. district court in Georgia (Case: Oldaker v. Giles. 2023). Women in ICE facilities are non-citizens who have been detained due to their immigration status. Detention facilities have been the site of serious and repeated allegations of abuse, with very low rates of legal representation (Featured Issue: Immigration Detention and Alternatives to Detention 2024). We focus on the issue of uninformed consent, both non-provision of key information and freedom from mis or dis-information. Initial allegations were raised by Project South, outlining a series of failures to meet basic medical needs of detainees during COVID-19. Amidst these broader complaints were reports of unnecessary hysterectomy procedures conducted at a concerning volume on detained women. Since the initial report, over a dozen detained women alleged they underwent a hysterectomy procedure at the Ocilla-based Irwin County detention center.

These surgeries (in which the uterus is removed) were either performed completely without consent, in the case of Mbeti Ndonga, or under the premise that the surgery was required for other clinical reasons. Gynecological experts, who reviewed the medical records of 19 detained women, shed light on the complex way in which uninformed consent manifested. For example:

“Ms. Castaneda-Reyes, stated that she was told she was having surgery to remove an ovarian cyst and that when she arrived for the surgery, an electronic tablet and a stylus were simply handed to her to sign with no explanation” (Ossoff and Johnson 2022)

Other detained women stated they were told they would “die” if they did not receive the surgery—indicating the severity of their clinical situation was exaggerated. Both represent cases of disinformation, or the intentional provision of incorrect information, regarding a clinical procedure (presumably) to induce consent.

### Norms and accountability

While signed consent forms for all women have not yet been identified, even amongst those who did provide signed consent, both US standards and international norms recognize that coercion can occur if consent was not informed. For example, ICE’s 2011 Performance-Based National Detention Standards define informed consent as: “An agreement by a patient to a treatment, examination, or procedure after the patient receives the material facts about the nature, consequences, and risks of the proposed treatment, examination or procedure; the alternatives to it; and the prognosis if the proposed action is not undertaken” (Operations Manual ICE Performance-Based National Detention Standards 2011). The Office of the High Commissioner for Human Rights (OHCHR) and six other UN agencies examine coercive or otherwise involuntary sterilization specifically in the context of human rights—outlining the aforementioned pre-conditions, including freedom from misinformation (The WHO 2014). They also specify that sterilization for prevention of future pregnancy cannot be justified on grounds of medical emergency. Within the Irwin detention center, clinical acuity and “medical emergency” were both used to indicate to patients that a hysterectomy was clinically necessary for reasons other than fertility control, despite medical records suggesting otherwise (Ossoff and Johnson 2022). Furthermore, detention itself has been characterized as an inherently a coercive setting where a person’s ability to give free, informed consent should be considered suspect; the onus on health care providers should be heightened (Sullivan et al. 2022).

### Broader prevalence

To explore the issue of uninformed consent beyond the above case, we look to a simple measure collected across countries, called the “Method Information Index” (MII). The MII is calculated from current contraceptive users’ responses to three questions on information given by a provider about their chosen contraceptive method. These questions do not capture all important aspects of information exchange, but they do make it possible to routinely measure and monitor some important elements of the information women receive (Track20 Country Data and Avenir Health. 2023), (p. 20) The MII for a method is estimated by the proportion of current users of that method who responded “yes” to all three questions: (1) were you informed about other methods, (2) were you informed about [your method’s] side effects and (3) were you told what to do if you experienced side effects (Jain 2016). This is understood as an underestimate of information required for consent i.e. the MII alone does not demonstrate whether a patient leaves their visit with a complete understanding of what to expect with their method (Chang et al. 2019).

**Fig. 1:**
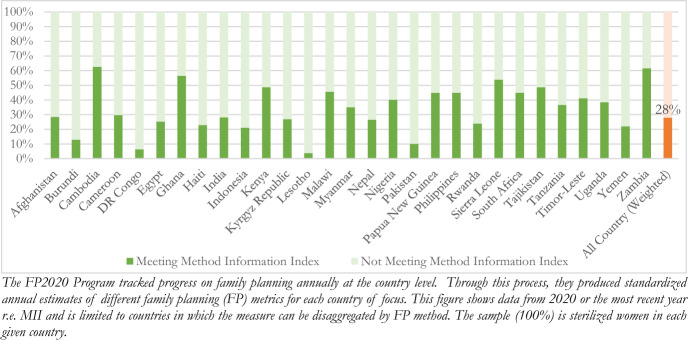
Share of sterilized women who are told about alternate options to sterilization & side effects of the procedure in 29 countries for which there is comparable data (2020)

As of 2020 Amongst the 29 countries with comparable data on the MII, only 28% of sterilized women met these basic criteria (Fig. 1). This aligns with prior research using data from 2000 to 2012, which found a lower MII was associated with socioeconomic variables—such as lower patient wealth (Jadhav and Vala-Haynes 2018). Comparing this rate to other family planning users e.g. those on the pill, using an IUD, etc. we find that sterilized women, on average, are statistically significantly less likely to get information required for informed consent (Appendix Table 3). These data are limited in that they represent a composite indicator made up of only three pre-conditions or pieces of information. However, in this context they raise more concern: not getting basic information required for consent is not only common, in almost every country examined, it was *more* common than receiving information (Table 1).

**Table 1:** Three recognized cases of coercive or otherwise involuntary sterilization care that can occur *with* patient consent

	Normative guidance^a^	Recognized case	Data r.e. less overt Coercion
Category I:^a^ uninformed consent	Adequate information provided to patient regarding procedure, side effects and permeance	ICE detention center hysterectomies	Cross-National Data on Method Information Index (MII)
	Freedom from misinformation (e.g. sterilization protects against HIV)		
Category II: incentivized consent	An individual must not be induced by incentives	India sterilization camp deaths	NFHS data on patient payment by facility type
	Provision or removal of social services dependent on receipt of sterilization		
Category III: constrained market consent	Alternate short and long-term options available to patient (alternatively referred to as “full choice”)	Uyghur long-term FP requirement	Cross-National Data on Herfindahl–Hirschman Index (HHI)

^a^Normative guidance adapted from the Cross-UN Interagency Statement on Eliminating Coercive and Otherwise Involuntary Sterilization Care, 2014; with specific human rights references cited throughout the piece as each category is raised

^b^Categories are illustrative, not comprehensive

## CASE II: Indian sterilization deaths

The second case we examine is a widely publicized case of sterilization-related deaths in India. Sterilization has a long and complex history in India (Singh et al. 2021). The government rolled out a mass-vasectomy programme in the late 1970s, which incited significant civil unrest (Connelly 2006; Gupte 2017). Following this period, the country transitioned to a near exclusive focus on tubal ligation (Basu 1985)—a procedure that can be conducted in outpatient settings by a range of medical professionals. As a result, contemporary sterilization care in India has been more integrated into the health system. However, in November of 2014, 83 women were sterilized over the course of a 1.5 hour period in Nemi Chand Jain Hospital (a non-operational, abandoned, facility) (Das 2014) Two days later, the same clinical team conducted 54 sterilizations at three other nearby sites in the central Indian state of Chhattisgarh (Arathi 2021). After being sterilized, at least 60 women began experiencing burning sensations, vomiting, abdominal pain and trouble breathing for which they were referred to two nearby hospitals (Arathi 2021). In rapid succession after arriving at the hospital, at least 13 of the admitted women died.

While the deaths occurred in hospitals, their surgical care was administered in what is commonly referred to as a “sterilization camp” or a fixed location in which multiple laparoscopic tubal ligations are conducted in succession—often by traveling physicians from a nearby city. While not always rural, many camps are intentionally run in areas with limited access to secondary or tertiary care. On the day of the camp, temporary medical infrastructure is set up (e.g. operating table, waiting wards, etc.) generally in a space that was not in use, sometimes school buildings on weekends, or in the case of Chhattisgarh, an abandoned hospital. The camps served as “pop-up” operating theaters, providing one service: female sterilizations. Certain aspects of the Chhattisgarh case were particularly striking: there was no running water in the abandoned hospital, 83 procedures were conducted in 1.5 hours, the clinical team used bicycle pumps to introduce air into women’s abdomens during procedures (generally done with an insufflator) (Mistreatment and Coercion 2018). While there are a multitude of issues in this case, we focus on a factor that may lead to a high volume of women consenting to sterilization care in unsafe or otherwise poor-quality facilities: incentives. Human Rights Watch and civil groups have raised issue that financial and non-financial incentives have often been provided in India for the receipt of sterilization and may encourage women to seek care in unsafe facilities, such as camps (India 2014).

### Specific norms and accountability

Both Indian case law and international human rights law outline incentives as problematic in the case of sterilization. The widely-publicized Chhattisgarh deaths informed an ongoing case in the Indian Supreme Court—Devika Biswas v. Union of India, which ultimately ruled to close all sterilization camps. In the ruling, the court stated:

“It is necessary to re-consider the impact that policies such as the… provision of incentives by the Government can have on the reproductive freedoms of the most vulnerable groups society whose economic and social conditions leave them with no meaningful choice in the matter and also render them the easiest targets of coercion” (Devika Biswas v. 2016).

Incentives are also outlined in the aforementioned U.N. Interagency Report, which states—individuals must not be induced by incentives (The WHO 2014). And further suggests the prohibition of public or private programs that provide incentives for patients to undergo sterilization. This is further reified in U.S. guidance for foreign aid. For example, the Tiahrt Amendment, which outlines pre-conditions for voluntarism within programs supported by USAID, states: “No incentives, bribes, gratuities, or financial reward for family planning program personnel for achieving targets or quotas, or for individuals in exchange for becoming a family planning acceptor.” (Voluntarism and Informed Choice. U.S. 2023). However, the government of India frames financial compensation for sterilization not as an “incentive” but rather the provision of financial support to cover costs patients incur when receiving the procedure i.e. akin to a conditional cash transfer.

### Data on prevalence

To explore the issue of incentivized consent beyond the Chandigarh case, we look at data collected from India’s nationally representative survey, the National Family Health Survey (NFHS-5) on payment to patents for sterilization by facility type. This data is collected from sterilized women at the household level. A limitation of this dataset is that we lack data on providers. In addition to payments made to patients, local motivators, members of India’s Accredited Social Health Activist (ASHA) or community health worker cadre, are often provided with financial compensation to identify women in the months leading up to a “camp day” that might be interested in receiving a sterilizing procedure. Using data collected from sterilized women, two variables are shown—the share of women in each facility type who were paid anything and the share of those women who were “Paid Above Net Neutral” or those who received more money than they spent (Table 2).

**Table 2:** Share of women paid for sterilization & share paid above net-neutral by facility type within a nationally representative sample in India

	Total sterilized population N = 188,569	By facility type				
		Sterilization camp N = 7,684	Public hospital N = 77,766	Private hospital N = 26,569	Community health center N = 49,699	Primary health center N = 15,761
Share of sterilized women paid	55.7%	74.8%	63.5%	5.5%	72.9%	68.2%
Mean amount received	₹ 3100 (SE: 46)	₹ 2532 (SE: 173)	₹ 3368 (SE: 73)	₹ 7500 (SE: 604)	₹ 2560 (SE: 69)	₹ 2920 (SE: 143)
Share above net neutral*	46.8%	27.5%	39.3%	95.0%	30.6%	34.1%

Estimates are generated using the NFHS-5 survey tool (applying the women’s individual sample weight, 6 decimals), which is a nationally representative survey that collects data at the household level regarding reproductive practices as well as location in which services were provided (i.e. facility type); facility types shown are the five most common locations in which women report having been sterilized

*Of those paid, share that were paid more than they expended to receive the procedure

The data on payment for sterilization by facility type suggest payment is widespread, not limited to compensation for costs incurred and not limited to camps. Indeed, camps had the lowest share of women paid more than they spent of all facility types at 27.5%. In the general sterilized population, nearly 50% of women who were paid were paid more than they spent to be sterilized—with nearly 40% of women in public hospitals (by far the most commonly reported site of sterilization care; 77,766 women) provided more than they spent.

Interpreting dollar/rupee amounts and the extent to which they are coercive is highly contextual (Różyńska 2022). Guidelines on eliminating coercive and otherwise involuntary sterilization refer to incentives broadly i.e. “*Individuals must not be induced by incentives*,” without specifying relative or absolute thresholds for what would constitute an incentive (The WHO 2014). Some ethicists might argue a cash transfer of any amount constitutes an incentive, while others consider contextual factors such as costs incurred while getting the procedure, annual wage and/or the existence of parallel incentive schemes for other forms of fertility control. Regardless, over 50% of sterilized women in India report having been paid. Absent critical assessment, all paid sterilizations are currently counted as “voluntary” procedures within both national and international metrics of modern family planning coverage.

## CASE III: *“Long Term Effectiveness” in Xinjiang*

The final case we examine is allegations of coercive sterilization amongst Uyghur women in Xinjiang, China. On June 26, 2020, a county-level government unit in Northern Xinjiang released a directive that stated: “married women of childbearing age who have adopted long-term birth control measures should be classified as ‘trustworthy personnel.’” This designation is in contrast to being deemed socially “untrustworthy” which can result in being sent to an internment camp (Byler 2022). Birth control methods include both sterilization and IUD insertion, but must be a long acting method. The policy both sets in place a contingency (in line with incentive-based consent discussed above), but also constructs a constrained “market” in which the methods available to women and deemed acceptable by the government are extremely limited.

### Norms and accountability

Beyond *knowledge* of alternate options, alternative options must also be made available in practice. The UN Interagency Report states this as a clear pre-condition for consent: “*There are alternative temporary methods of contraception, including long- and short-term methods*” (The WHO 2014). Less overt forms of coercion may be intersecting and overlapping—particularly in contexts that involve detention or otherwise constrained mobility. For example, in addition to having an intentionally constrained market (family planning limited to long-acting options) human rights reports suggest Uyghur women in Xinjiang are also incentivized to report community members who do not adhere to birth-control guidelines and undergo regular, unannounced, house visits.

The situation in Xinjiang represents constrained market choice both in terms of what is physically made available, but also in terms of what is deemed legally permissible by the government. The policy includes both surgical sterilization (either hysterectomy or tubal ligation) as well as long-acting reversable contractive (LARCs) such as IUDs which, absent viable removal options, have been characterized as “functional sterilization” from both a practical and ethical perspective (Tännsjö 2006). Recent evidence suggests women often face barriers in getting long-term provider-dependent methods removed—which aligns with other bioethics accounts that place IUD (with limited options for removal) and sterilization in the same category ethically (Tännsjö 2006; Britton et al. 2021).

### Data on prevalence (HHI)

**Fig. 2:**
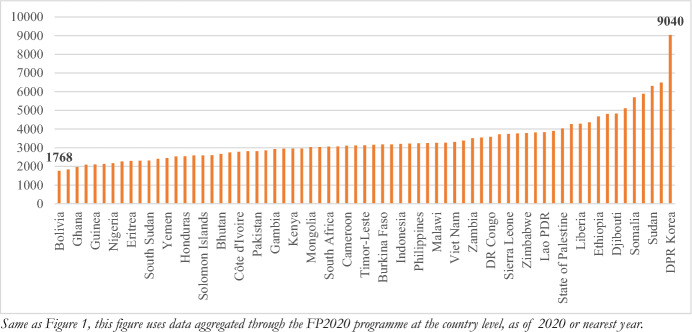
Herfindahl–Hirschman Index Assessing Family Planning Market Diversity (2020)

To examine constrained-market consent, we generate a measure of market concentration—the Herfindahl–Hirschman Index. This allows us to examine the distribution of mFP methods in use. This measure uses data from Family Planning 2020 Indicator 9 “Method Mix” to create a single variable of concentration using data on the distribution of mFP methods in use within a given country. In line with methodology used to study insurer concentration, HHI values range from 0 to 10,000, with an HHI closer to zero indicating a more “competitive” healthcare market and closer to 10,000 indicating a less competitive market (KFF 2021). Here, each method is treated as a “market actor” which generates a single, indirect, measure of available choice or market diversity. A limitation of this approach is that data is only available for methods currently *in use*—so while it sheds light on if a market might be constrained, it does not allow us to disentangle patient preference (Fig. 2).

A stark example is the Democratic People’s Republic (DPR) of Korea, which has the highest contraceptive prevalence rate of any focus country (54%) yet, by far, the most concentrated family planning “market” with a HHI of 9,040 (highly concentrated) where over 95% of modern family planning users are utilizing an inter-uterine device (IUD). Looking at market concentration in isolation does not allow for the direct assessment of coercion, for example: women in DPR Korea *may* have a higher baseline preference for IUDs than individuals in other countries. However, the high rate of method concentration raises concern, particularly regarding choices at the point of purchase and availability of alternate options.

## Discussion

Sterilization is a unique case within the broader landscape of coercion and public health practice—there are clear and internationally agreed upon human rights norms and frameworks, as well as bioethical ones (Vereinte Nationen 2022). These norms are comprehensive and nuanced—recognizing the many ways in which coercion can manifest, including in the absence of overt force as well as when patients technically do provide consent (WHO 2014). As such, there is definitional clarity including the context in which care is provided and broader structural factors that may constrain choice. What has been less clear is how these definitions should manifest in practice. The data explored in this piece suggest that if we take rights-based guidance seriously, coercion within sterilization care may be more prevalent and normalized than is often understood.

An empirical approach, while limited, is useful. It allows us to assess coercion or threats to consent, as defined through normative rights frameworks. For example, with the data presented above, we can see that “uninformed consent” our first category of interest, is not limited to overt instances—such as the ICE detention center. In fact, it appears it may be the status quo to undergo a sterilizing procedure without basic information required for informed consent, at least within the handful of countries in which we have comparable data. Similarly, while reports of egregiously unsafe physical conditions raised concern regarding India’s sterilization camps, a more pervasive and less overt issue may be payment. Financial incentivization is common across facility types. Given the majority of sterilizing procedures are conducted public hospitals and community health centers, this raises concern regarding induced choice both within and beyond the camp context, a recognized form of manipulation in the bioethics literature and a recognized form of coercion in the human rights literature (Faden 1986; The WHO 2014). It also suggests one of the primary approaches to address coercive practices—closing sterilization camps—may have limited impact (Devika Biswas v. Union of India. International Network for Economic, Social Cultural Rights. Published 2016).

Ensuring the international normative frameworks surrounding coercion translate into practice requires, at the very least, states to enact enabling legal and policy frameworks that allow for the identification of coercion. International norms are clear that sterilizations should only be conducted in a manner that respects the human rights and dignity of the individual and are free from coercion (The WHO 2014). Moreover, health care providers must respect the outcome—that is the choices of individuals and should not pressure them towards using one contraceptive method over another, or none (Senderowicz 2020). For that reason, it is important that there is monitoring and oversight of the contexts in which people are asked to make choices regarding sterilization, as well as accountability for any violations (The WHO 2014). In the case of the ICE hysterectomies, the “Ossoff Report” commissioned by U.S. members of Congress found that ICE technically did not have a responsibility to monitor informed consent because providers are professionally and legally obligated to ensure informed consent (Ossoff and Johnson 2022). Similarly, in many countries, sterilizations are categorized as a modern family planning method; patients undergoing sterilization are aggregated with those on other forms of family planning, all of whom are assumed to be consenting.

This issue, of assumed consent, is relevant to problematize moving forward. Apart from legal redress, we lack accountability mechanisms for more routine violations. In their absence, adherence to human rights guidelines is assumed. The motivation for maintaining this status quo is multifaceted. One significant factor may be the existence of several restrictions on U.S. foreign aid that prohibit the administration of funds to entities engaged in “coercive” practices (Voluntarism and Informed Choice. U.S. 2023). Restrictions, while well intentioned, may have the adverse effect of disincentivizing candid reporting which would compromise receipt of aid. Regardless of cause, the data presented in this piece, which surface the potential prevalence of less overt, and potentially more routine, forms of coercion raise the ethical implications of *not* accounting for coercion. While coercion is conceptually complex—even looking at three cases, taking a functional approach to assessment allows us to see that patient’s experiences with care may differ drastically from government reporting. Specifically, all governments that signed onto the aforementioned ICPD or Cairo Platform of Action agreed to end population policies that utilized coercion and instead exclusively support voluntary family planning programs (Langer 2006). However, aspects of coercion that involve structural manipulation (such as financial payment, restricting the market, etc.) appear quite common when assessed using data collected from patients themselves i.e. aggregated FP2020 indicators and the Indian household survey.

This discordance, and the relevance of a functional approach to measurement, is a relevant approach across public health. For example, when the U.S. began assessing usage of Electronic Records (EHRs) across the country—they employed a survey in which hospital representatives were asked a simple binary question: “do you have an EHR?” Almost all groups answered in the affirmative—they had computers. In 2009, researchers fielded a survey in which representatives were asked about a series of functionalities which, taken together, constituted an EHR; for example: clinical documentation, test and imaging results, computerized provider-order entry, etc. (Jha et al. 2009). The responses differed dramatically from the previously ubiquitous “yes”, providing both a more comprehensive and actionable assessment of EHR usage across the country. A parallel situation may be occurring with coercive sterilization care. If asked directly if coercion exists, the answer will likely be an understandable and unambiguous “no” —governments, providers and policymakers are aware that coercion is normatively unacceptable and counter to both human rights norms and national clinical guidelines. If, however, we take a functional approach to assessing coercion and pose questions regarding its component parts—we may get a dramatically different response.

It is also possible there is no malintent on the part of the providers, who also operate within constrained markets. However, coercion can still occur functionally, regardless of intent. The Istanbul Report, for example, specifies that"passive"violations of rights by health professionals still constitute violations."(Vereinte Nationen 2022)^3^ While health professionals have a duty not to participate in torture or ill-treatment practices, and to document and report such practices (including forced and coercive sterilization), violations may occur unbeknownst to the provider. This is particularly true if violations are more subtle or routine, such as a lack of alternate options, and may not present as a rights violation to a provider who is operating with limited options on the “supply” side of the healthcare interaction. If anything, this highlights the importance of broader structural mechanisms of accountability beyond any individual, patient or provider.

The implications, particularly for sterilization care, are stark. The fact is—there is currently no effort to routinely assess coercion within public health practice at the population level in a meaningful way. If we conceive of coercive or otherwise involuntary sterilization as rare, then isolated legal adjudication is an adequate and appropriate means of accountability. However, as we outline in this piece, even a limited empirical assessment of pre-conditions for consent raises concern. In contrast, measures of volume-based coverage are actively assessed and lauded. Increasing the absolute number of women utilizing a modern family planning method was the organizing goal for Family Planning 2020 (FP2020), and remains a focus of many development programs (Brown et al. 2014). Absent accounting for coercion, all sterilized women (regardless of informed consent) are currently counted as a “success” within key development indicators of reproductive coverage. Not only is sterilization a recognized form of modern family planning, it is the most common form of family planning utilized in the world (UNDP 2020). Understanding if people choosing sterilization do so freely, with full information, is essential to ensure this common surgical procedure is performed both legally and ethically.

## Data Availability

Data utilized in this paper are from (1) FP2020 Track20 Annual Reporting, which aggregates multiple national data sources on family planning at the country-level for 70 countries, and, (2) India-specific data is from the National Family Health Survey, Round 5 (NFHS-5). All data are de-identified and publicly accessible. The NFHS-5 requires a non-monetary application through the Demographic and Health Survey (DHS) Program dataset site, which is operated by USAID.

## Appendix

See Table 3.

**Table 3:** Wilcoxon Signed Rank Sum Test—MII amongst sterilized v. all modern family planning users

	Observed	Expected	Significance level	
			One-sided tests	Two-sided test
Positive	6	15	0.9998	0.0014
Negative	24	15	0.0007	
Zero	0	0		

*Sterilized Women: This is an aggregated measure of women who report using sterilization as a family planning method in a given country; this may include women who have undergone a tubal ligation procedure or hysterectomy

All mFP Users: This measure also includes sterilized women due to an inability to remove this population from the aggregated data.

Looking at the results of the Wilcox test to assess for difference, there is a statistically significant difference in information provided to sterilized women versus the general population of mFP users. The p-values displayed in Appendix Table 3 are based on a binomial distribution for the test statistic, which is the number of positive or negative signs. The n for the test is the number of nonzero differences. Because no approximation is used, the p-values are “exact” p-values. The p-value for the one-sided test, where the alternative hypothesis is that the median of the sterilized population—all modern family planning users is smaller than zero, is 0.0007. The p-value for the two-sided test, where the alternative hypothesis is simply that the median of the differences is different from zero, is 0.0014; both falling well below a 0.05 threshold.

## Footnotes

1 Cairo, Para 7.3; Beijing paras. 95 and 96.

2 General Recommendation No. 24, Women and Health, UN Doc. No. CEDAW/C/1999/I/WG.II/WP.2/Rev.11999 para. 20. Note also, the Convention on the Rights of Persons with Disabilities requires States Parties to protect this population against non-consensual experimentation as well as recognize their equal capacity to consent. Convention on the Rights of Persons with Disabilities, G.A. Res, 61/1062006, arts. 12, 15, 16, 17 and 25.

3 A/HRC/31/57; A/HRC/7/3, paras. 25–26; and OHCHR, “Gender-based crimes through the lens of torture International Women’s Day”, press release, 8 March 2016.
